# Unraveling the Anti‐Obesity Potential of White Kidney Bean α‐Amylase Inhibitors: Mechanistic Insights From Enzyme Kinetics to Gut Microbiota Modulation

**DOI:** 10.1002/fsn3.71043

**Published:** 2025-10-02

**Authors:** Jiai Yan, Jianguang Zhao, Pamila Naizemuding, Wei Zhao, Jing Sun, Yingyu Wang, Ju Yang, Dan Li, Feng Zhang, Hong Cao

**Affiliations:** ^1^ Affiliated Hospital of Jiangnan University Wuxi Jiangsu People's Republic of China; ^2^ Wuxi School of Medicine Jiangnan University Wuxi Jiangsu People's Republic of China; ^3^ Suzhou Langbang Nutrition Co. Ltd Suzhou Jiangsu People's Republic of China; ^4^ China Mengniu Dairy Company Limited Shanghai People's Republic of China; ^5^ School of Food Science and Technology Jiangnan University Wuxi Jiangsu People's Republic of China; ^6^ Yixing Institute of Food and Biotechnology Co. Ltd. Yixing China

**Keywords:** α‐amylase inhibitors, enzyme kinetics, gut microbiota, obesity, white kidney bean

## Abstract

The global rise in obesity, driven largely by excessive carbohydrate consumption, highlights the demand for innovative dietary interventions targeting starch digestion. This study investigates the anti‐obesity effects of α‐amylase inhibitors (α‐AI) extracted from white kidney beans, employing a multidisciplinary strategy encompassing botanical screening, enzyme kinetics, clinical trials, and gut microbiota profiling. Among 10 varieties evaluated, the A10 strain from Jilin Province demonstrated the highest α‐AI activity, characterized by noncompetitive inhibition that remains effective across varying starch concentrations. In an 8‐week randomized controlled trial, α‐AI supplementation significantly reduced body weight, BMI, waist circumference, and hip circumference compared to placebo. Further, 16S rRNA sequencing revealed dual mechanisms: enrichment of SCFA‐producing bacteria (e.g., *Bifidobacterium* and *Bacteroides ovatus* ) and modulation of microbial lipid metabolic pathways. These results highlight α‐AI as a dual‐action anti‐obesity agent, combining direct enzymatic inhibition with microbiome‐mediated metabolic effects. By bridging phytochemical characterization with clinical outcomes, this work proposes a novel therapeutic approach that simultaneously targets carbohydrate absorption and gut microbial ecology, supporting the development of standardized α‐AI formulations as potential nutraceuticals for metabolic syndrome.

## Graphical abstract

This study demonstrated that α‐amylase inhibitors (α‐AI) from white kidney beans act as non‐competitive inhibitors, maintaining efficacy across starch concentrations. α‐AI significantly reduced obesity markers and exerted dual mechanisms: enriching SCFA‐producing bacteria like *Bifidobacterium* while modulating microbial lipid metabolism. These findings highlight a novel approach that simultaneously targets carbohydrate absorption and gut microbial ecology, supporting α‐AI's potential as a nutraceutical for metabolic syndrome.

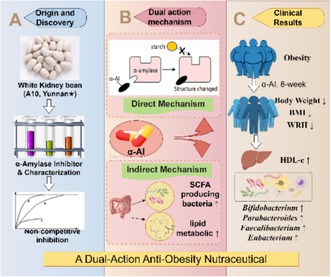

## 1 Introduction

The global obesity epidemic has emerged as a critical public health challenge, with high‐carbohydrate diets identified as a significant contributor to excessive caloric intake and metabolic dysregulation (Zhao et al. 2017). Epidemiological studies have demonstrated a significant correlation between the rising prevalence of obesity and increased carbohydrate consumption (Austin et al. 2011; Tobias et al. 2015). Rathnayake et al. (Rathnayake et al. 2014) suggested that high‐carbohydrate diets and physical inactivity may explain the high prevalence of central obesity among Sri Lankan women. Furthermore, dietary carbohydrate intake has been associated with metabolic syndrome in both Korean men and women (Song et al. 2014). In China, the traditional diet remains predominantly rice/wheat‐based and high in carbohydrates, which can lead to excess energy intake and an increased risk of obesity (Batis et al. 2014). Modifying dietary patterns to reduce carbohydrate intake may be an effective strategy for decreasing the prevalence of obesity.

To address this issue, researchers are investigating various strategies to regulate carbohydrate intake. In this context, bioactive plant‐derived compounds have garnered considerable interest and are increasingly utilized in areas such as functional biomaterials and small‐molecule therapeutics (Peng et al. 2021; Guo, Guo, et al. 2024; Bai et al. 2025; Ma et al. 2025; Zhang et al. 2025). Among them, the white kidney bean ( *Phaseolus vulgaris* L.) extract—a potent source of α‐amylase inhibitor (α‐AI)—has drawn significant attention for its ability to inhibit the enzymatic breakdown of starch into absorbable glucose (Udani and Singh 2007). The content and activity of inhibitors in white kidney beans are closely associated with the raw materials utilized (Chinedum et al. 2018). Consequently, the selection of appropriate raw materials from white kidney beans has emerged as a central focus of this study. Empirical evidence indicates that the white kidney bean inhibitor can mitigate starch breakdown by inhibiting α‐amylase activity, thereby reducing postprandial blood glucose levels and overall energy intake (Qin et al. 2019; Wang et al. 2021). Clinical trials corroborate these findings: a 12‐week randomized controlled trial involving 81 overweight Chinese adults demonstrated that a daily intake of 1000 mg of white kidney bean extract resulted in reductions in body weight, fat mass, BMI, waist circumference, and hip circumference (Jäger et al. 2024). Mechanistically, this weight loss is attributed not only to caloric restriction but also to enhanced insulin sensitivity (Argyrakopoulou et al. 2020).

The gut microbiota plays a crucial role in the development and progression of obesity. Studies have shown that the composition and diversity of the gut microbiota are closely linked to obesity (Liu, et al. 2025). By regulating the gut microbiota, it is possible to effectively control obesity and its associated metabolic disorders (Cao et al. 2024). Dietary fiber can improve the balance of the gut microbiota, thereby reducing the incidence and severity of obesity, where microbial fermentation generates short‐chain fatty acids (SCFAs) (Zhao et al. 2018). Emerging animal evidence highlights the dual role of white kidney bean inhibitors in metabolic and gut microbiota modulation (Chen et al. 2023). However, existing research has not integrated white kidney bean inhibitors from raw material selection, enzyme kinetics studies, to clinical weight control.

This study aims to address these gaps through a comprehensive investigation of white kidney bean α‐amylase inhibitors. We first optimized raw material selection and α‐AI activity quantification protocols. The inhibition type and starch concentration‐dependent effects were mechanistically characterized in vitro. Subsequently, a randomized controlled trial evaluated the extract's effects on body weight, lipid metabolism (triglycerides, LDL‐c, HDL‐c), and gut microbiota composition. Our findings offer new insights into the multi‐targeted anti‐obesity mechanisms of α‐AI and lay the groundwork for the standardized development of nutraceuticals.

## 2 Materials and Methods

### 2.1 Materials

**FIGURE 1:**
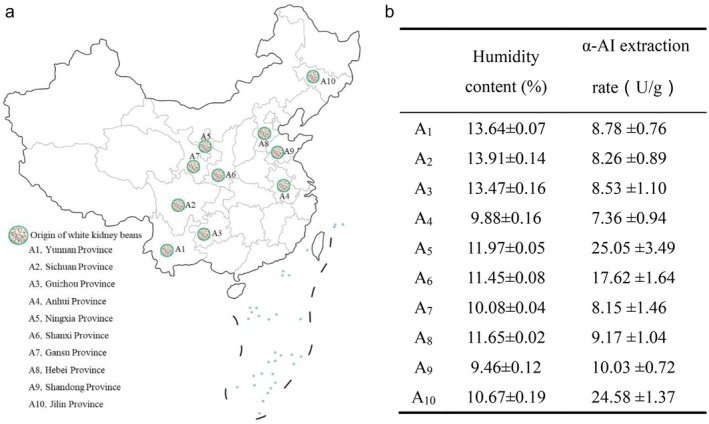
Origin of white kidney beans and humidity content and extraction rates of α‐AI in 10 types of white kidney beans. (a) The origin of 10 types of white kidney beans; (b) humidity content and extraction rates of α‐AI in 10 types of white kidney beans (Date are mean ± SD).

Eight types of white kidney beans were used, all commercially available. The places of origin are Yunnan Province (A1), Sichuan Province (A2), Guizhou Province (A3), Anhui Province (A4), Ningxia Province (A5), Shanxi Province (A6), Gansu Province (A7), Hebei Province(A8), Shandong Province(A9), and Jilin Province (A10) (Figure 1). The beans are full, have a regular shape, and show no signs of pests, diseases, or mechanical damage.

Pig pancreatic α‐amylase, bovine trypsin, and pepsin BApNA; 2,4,6‐trinitrobenzenesulfonic acid (TNBS) and Folin phenol reagent: Sigma Aldrich, USA; flavor protease, neutral protease, and alkaline protease: Novozymes Biotechnology Co. Ltd.; Prote AX and Protease N “Amano” G (hereinafter referred to as “Amano”): Amano Enzyme Products Co. Ltd., Japan; 3,5‐dinitrosalicylic acid (DNS), soluble starch, tyrosine, L‐leucine, dimethyl sulfoxide, casein, sodium dodecyl sulfate (SDS), and other reagents are all analytical grade: China National Pharmaceutical Group Chemical Reagent Co. Ltd.

### 2.2 Selection of White Kidney Bean Varieties

After individually processing 10 varieties of white kidney beans (Labeled A1–A10), the resulting powder was passed through a 60‐mesh sieve. The sieved flour was then mixed with deionized water in a 1:5 mass‐to‐volume ratio and subjected to extraction for 1 h at ambient temperature. Post‐extraction, the mixture underwent centrifugation at 8000 rpm for 30 min at 4°C to isolate the supernatant (Wang et al. 2011). Any residual particulates in the supernatant were removed using a 120‐mesh sieve, resulting in the production of the white kidney bean aqueous extract. The activities of α‐AI within the extract were quantified, and the extraction efficiencies (U/g) of α‐AI from the different bean varieties were determined and compared. One unit of α‐amylase inhibitor activity (U) is defined as the amount of inhibitor required to suppress the generation of 1 μg of maltose per minute under the assay conditions (37°C, pH 6.9) in a reaction system containing 100 U of α‐amylase. This analysis facilitated the identification of the most suitable white kidney bean variety for further experimentation. The extraction efficiency of α‐AI was calculated based on the activity of these compounds extracted per gram of bean powder.

### 2.3 Determination of α‐AI Activity

The inhibitory activity of α‐amylase was measured as described in the study by Bernfeld with slight modifications (Bernfeld 1955). Add 0.25 mL of α‐amylase solution (1.5 U/mL) and 0.25 mL of sample to 0.5 mL of PBS, and after 10 min in a water bath at 37°C, add 0.5 mL of soluble starch solution (1%). After precise reaction for 5 min, add 1 mL of DNS reagent to terminate the reaction. Heat the reaction solution in a boiling water bath for 10 min and quickly cool it to room temperature in an ice water bath. Then add 5 mL of deionized water, mix well, and measure the absorbance at a wavelength of 540 nm. During the measurement process, blank tubes, blank control tubes, and inhibitory control tubes are set up. No sample is added to the blank tube, no α‐amylase solution or sample is added to the blank control tube, and no α‐amylase solution is added to the inhibition control tube. All areas with insufficient volume are supplemented with PBS. The measurement system is shown in Table 1.

**TABLE 1:** System for measuring α‐AI activity.

	Enzyme (mL)	Sample (mL)	PBS (mL)	Starch solution (mL)	DNS (mL)	DD water (mL)
Blank tube	0.25	—	0.75	0.5	1	5
Blank control	—	—	0.75	0.5	1	5
Inhibition tube	0.25	0.25	0.5	0.5	1	5
Inhibition control	—	0.25	0.75	0.5	1	5

The inhibition rate (AR, %) of α‐AI on α‐amylase in the sample is calculated according to the following formula:

(1) $$\mathrm{AR}=\left(1-\frac{A3-A4}{A1-A2}\right)\times 100\%$$

Note: In the Formula (1), A1, A2, A3, and A4 represent the absorbance values of blank tube, blank control tube, suppression tube, and suppression control tube at 540 nm, respectively.

The α‐AI activity (U) in the sample is calculated according to the following formula:

(2) α−AIactivity=AR×1.5×n×V

Note: In the Formula (2), 1.5 represents activity of α‐amylase solution (U/mL), *N* represents dilution factor of the sample, and *V* represents the total volume of the sample (mL).

### 2.4 Determination of α‐AI Inhibition Types in White Kidney Beans

The measure of α‐AI inhibition types was according to Shamim et al. (Shamim et al. 2020) with slight modifications. Prepare a 10 mg/mL inhibitor solution of the extract of white kidney bean α‐AI using PBS (pH 6.9). After incubating α‐amylase solutions of different concentrations (0.0125, 0.025, and 0.05 mg/mL) with inhibitor solutions or PBS at 37°C for 10 min, the reaction rate (mg/min) of soluble starch with a catalytic concentration of 10 mg/mL catalyzed by inhibited α‐amylase and non‐inhibited α‐amylase was measured. The reaction rate was calculated as the amount of glucose generated per minute (mg). Plot the enzymatic reaction kinetics graph with α‐amylase concentration as the *x*‐axis and reaction rate as the *y*‐axis.

After incubating 0.025 mg/mL of α‐amylase solution with inhibitor solution and PBS at 37°C for 10 min, the reaction rates of inhibited α‐amylase and non‐inhibited α‐amylase catalyzing soluble starch solutions of different concentrations (5, 10, 15, 20 mg/mL) were measured. Draw a Lineweaver–Burk curve with 1/*S* as the horizontal axis and 1/*V* as the vertical axis.

### 2.5 Effect of Starch Concentration on the Inhibition of α‐AI From White Kidney Bean

This method was derived from the works of Ma et al. (Ma et al. 2018) Prepare a 2 mg/mL inhibitor solution of the extract of white kidney bean α‐AI using PBS (pH 6.9). In the process of measuring inhibitory activity, soluble starch solutions of different concentrations (0.25%, 0.5%, 1%, 1.5%, 2%, m/v) were added, and other conditions were kept constant to determine the inhibition rate of white kidney bean α‐AI on α‐amylase at different starch concentrations.

### 2.6 The Effect of Starch Type on the Inhibition of α‐AI From White Kidney Beans

Prepare 1% (m/v) starch solutions by separately dissolving potato starch, rice starch, wheat starch, and corn starch in water. In the process of measuring inhibitory activity, potato starch solution, rice starch solution, wheat starch solution, and corn starch solution were used instead of soluble starch solution, while keeping other conditions unchanged. The inhibition rate of white kidney bean α‐AI on α‐amylase was measured under different types of starch solutions (Ma et al. 2018).

### 2.7 Clinical Trial Design and Participants

The parallel‐controlled 8‐week trial was performed from January 2018 to November 2018 in the Third Affiliated Hospital, Nantong University (renamed later as the Affiliated Hospital of Jiangnan University), Wuxi, Jiangsu, China. The trial was approved by the ethical review board of the Third Affiliated Hospital, Nantong University (ID: IEC201711001) and registered on the Chinese Clinical Trial Register as ChiCTR‐IOR‐17013656. Written informed consent was obtained from all participants before the intervention. Randomization was implemented using the PLAN procedure in SAS software. Participants, healthcare providers, and outcome assessors were blinded to group assignment. Identically appearing α‐AI extract and placebo (maltodextrin) were manufactured and packaged by an independent company. The blinding code was held by a doctor who was not involved in participant recruitment or intervention and was not broken until all data analyses were completed.

This study was a component of a larger clinical trial (Registration number: ChiCTR‐IOR‐17013656) investigating the efficacy of α‐AI‐based intervention for weight management in obese participants with type 2 diabetes. Twenty‐six volunteers aged 35–65 years with type 2 diabetes and obesity (BMI ≥ 24) were selected to evaluate the effects of white kidney bean extract on weight control. Eligibility criteria included Han ethnicity, local residency for more than 5 years, BMI ≥ 24, and HbA1c levels between 6.5% and 13.0%. Apart from sulfonylureas and insulins, most hypoglycemic agents have been reported to modulate gut microbiota (Liu et al. 2022). Therefore, this study included only patients treated with sulfonylureas or insulin. Patients were excluded if they had type 1 diabetes, malignant hypertension, severe cardiac disease, renal failure (eGFR < 15), kidney replacement, inflammatory bowel disease, gastrointestinal ulcer, an autoimmune disease, thyroid/pituitary dysfunction, severe diarrhea, or cancer. Exclusion criteria also included patients receiving medication or surgery for losing weight within 3 months, receiving the administration of antibiotics within 1 month, receiving gastrointestinal surgery, or during or preparing for pregnancy and lactation. Subjects with poor compliance or protocol violation or unwillingness to continue the clinical trial were asked to withdraw from this study. Participants maintained their usual diet and exercise routines during the study. In the end, 24 participants were eligible and decided to participate in this study (the baseline was shown in Table 2).

**TABLE 2:** Baseline characteristics of study participants.

Basic indicators	Group C (*n* = 12)	Group T (*n* = 12)	*p*
Age (years)	57.50 ± 1.69	56.83 ± 1.63	0.795
Sex (M/F)	3/9	4/8	0.917
BMI (kg/m^2^)	26.28 ± 0.60	26.10 ± 0.51	0.812
Body weight (kg)	67.23 ± 1.94	66.86 ± 1.59	0.888
Hip circumference (cm)	92.42 ± 1.61	90.67 ± 1.23	0.418
Waist circumference (cm)	99.08 ± 1.27	99.83 ± 0.62	0.619
waist to hip ratio (WHR)	0.93 ± 0.01	0.91 ± 0.01	0.125
HbAlc (%)	7.63 ± 0.19	7.29 ± 0.19	0.253
TG (mmol/L)	4.99 ± 0.33	4.95 ± 0.18	0.928
HDL‐C (mmol/L)	1.14 ± 0.07	1.41 ± 0.16	0.166
LDL‐C (mmol/L)	3.09 ± 0.23	2.99 ± 0.28	0.776

*Note:* Date are mean ± SD (standard deviation).

All subjects were randomly assigned in a 1:1 ratio to the control group (Group C, receiving interference from maltodextrin, 1.5 g/day) and the T group (Group T, receiving interference from α‐AI extracted from White Kidney Bean, 1.5 g/day (α‐AI activity, 30 000 U), raw material preparation technology provided by Suzhou Langbang Nutrition Co. Ltd). After enrollment, demographic data, including sex, age, disease history, medication, and lifestyle, of all subjects were recorded. Anthropometric data, including body mass index (BMI) and body weight, were collected. A priori, the primary endpoint of this study was defined as the change (Δ) in body mass index (BMI) from baseline to Week 8. Secondary endpoints included changes in body weight, waist circumference, lipid profile (including TG, LDL‐C, and HDL‐C), and other metabolic parameters.

The change in the primary outcome, BMI, was detected during the intervention. Secondary outcomes included changes in the levels of lipid metabolism and fecal microbiota. Outcomes representing lipid metabolism included changes in the levels of triglycerides (TG), high‐density lipoprotein cholesterol (HDL‐c), and low‐density lipoprotein cholesterol (LDL‐c).

### 2.8 16S rRNA Sequence Detection

Fecal bacterial DNA extraction, NGS library preparation, and sequencing were performed at Shanghai HonsunBio Technology Co. Ltd. (Shanghai, China).

#### 2.8.1 DNA Extraction, PCR Amplification, and Sequencing

Microbial DNA was extracted from fecal samples using an E.Z.N.A. soil DNA Kit (Omega Bio‐tek, Norcross, GA), according to the manufacturer's protocols. The NanoDrop 2000 UV–vis spectrophotometer (Thermo Scientific, Wilmington, Delaware, United States) was used to determine the final DNA concentration and purity, and DNA quality was checked using 1% agarose gel electrophoresis. The V3–V4 hypervariable regions of the bacterial 16S rRNA gene were amplified with the primers 338F (5′‐ACTCCTACGGGAGGCAGCAG‐3′) and 806R (5′‐GGACTACHVGGGTWTCTAAT‐3′) by a thermocycler PCR system (GeneAmp 9700, ABI, Foster, CA, United States). The PCR reactions were as follows: denaturation at 95°C for 3 min, 27 cycles of denaturation at 95°C for 30 s, annealing at 55°C for 30 s, elongation at 72°C for 45 s, extension at 72°C for 10 min, and ending at 4°C. Each 20 μL reaction mixture contained 4 μL of 5× TransStart FastPfu buffer, 2 μL of 2.5 mM dNTPs, 0.8 μL of each primer (5 μM), 0.4 μL TransStart FastPfu DNA Polymerase, and 10 ng template DNA. PCR was performed in triplicate. The PCR product was extracted from 2% agarose gel and purified using an AxyPrep DNA Gel Extraction Kit (Axygen Biosciences, Union City, CA), according to the manufacturer's instructions, and quantified using Qubit 4 (Thermo Fisher, United States). Purified amplicons were pooled in equimolar concentrations and paired‐end sequenced on the Illumina MiSeq PE300 platform (Illumina, San Diego, United States) according to the standard protocols by Honsunbio Technology Co. Ltd. (Shanghai, China).

#### 2.8.2 Bioinformatics Analysis

Sequencing reads were demultiplexed, quality controlled by fastp (version 0.21.0), and merged by FLASH (version 1.2.7). Shortly, reads with adaptor sequences and low‐quality bases (quality score < Q20) were trimmed. Truncated reads shorter than 50 bp and reads containing ambiguous nucleotides were discarded. Subsequently, the paired‐end reads were merged according to the minimum overlap of 10 bp with a maximum mismatch ratio of 0.2 in the overlapping region. Only merged sequences were retained for downstream analyses. The UPARSE algorithm was used to cluster sequences with a 97% similarity cutoff, while chimeric sequences were identified and removed. Next, the taxonomy of each OTU representative sequence was assigned by using RDP Classifier against the reference database SILVA138 with a minimum confidence score of 0.7. Rarefaction was performed in order to compare the abundance of OTUs across samples.

Sequences demultiplexed were imported to QIIME2 (version 2022.8). The DADA2 algorithm was used to quality filter and denoise sequences, while chimeric sequences were removed. Next, the taxonomy of each ASV representative sequence was assigned by using RDP Classifier against the reference database SILVA138 with a minimum confidence score of 0.7. The number of sequences from each sample was normalized to the lowest number of read counts by randomly selecting subsets of sequences.

### 2.9 Statistical Analysis

The R (version 4.1.3) was used to perform general statistical analysis and visualize results via packages vegan (v2.6–4), phyloseq (v1.38.0), tidyverse (v1.3.2), ggpubr (v0.5.0), ComplexHeatmap (v2.10.0), and corrplot (v0.92). Alpha diversity was estimated using the Sobs, ACE, Chao1, Shannon, and Simpson indices. Principal coordinates analysis (PCoA) based on bray‐curtis matrices, with statistical significance determined by permutational multivariate analysis of variance (PERMANOVA), was conducted to assess the differences in beta diversity between groups. For comparing the relative abundance of different taxa between groups, linear discriminant analysis (LDA) effect size (LEfSe) method was performed with a *p*‐value < 0.05 for the Kruskal–Wallis test and a size‐effect threshold of 2.0 on the logarithmic LDA score. Spearman's rank correlation analysis was used for correlation analysis.

Prism 8 (GraphPad Prism, San Diego, CA) statistical software was used to analyze the data. A two‐way repeated‐measures analysis of variance (ANOVA) with a mixed effects model was used for intra‐ and intergroup comparisons. The normality of all continuous data was confirmed using the Shapiro–Wilk test (*p* > 0.05). Homogeneity of variances was assessed using Levene's test (*p* > 0.05). As all assumptions were met, between‐group differences for continuous variables were analyzed using the independent samples *t*‐test. Statistical tests were two‐sided, and a *p* value < 0.05 was considered to be statistically significant.

## 3 Results and Discussion

### 3.1 Determination of the Optimal Variety of White Kidney Beans

Figure 1b compares the humidity content and extraction rates of α‐AI in 10 types of white kidney beans. There was no significant difference in humidity content among different types of white kidney beans, with humidity content ranging from 9.46% to 13.91%. The difference in the extraction rate of α‐AI was most significant. The α‐AI extraction rates of white kidney beans A5 and A10 were the highest, at 25.05 U/g and 24.58 U/g, respectively, significantly higher than those of other types of white kidney beans (*p* < 0.05). Nevertheless, A5 exhibited a greater humidity content compared to A10, and the output of white kidney beans in Ningxia Province is comparatively limited. Therefore, considering the physiological activity and sustainability of production of the white kidney bean α‐AI preparation, white kidney bean A10 was determined as the optimal white kidney bean variety and used for subsequent experiments. This selection was based not only on high α‐AI activity but also on key practical considerations. The lower moisture content of A10 suggests better stability during storage, reducing risks of degradation (Damodaran et al. 2008). Moreover, its superior yield profile in Jilin ensures a more reliable and sustainable supply chain for potential future scale‐up, a critical factor often highlighted in bioprocessing research (Guo, Liu, et al. 2024).

### 3.2 Inhibition Types of α‐AI From White Kidney Beans

**FIGURE 2:**
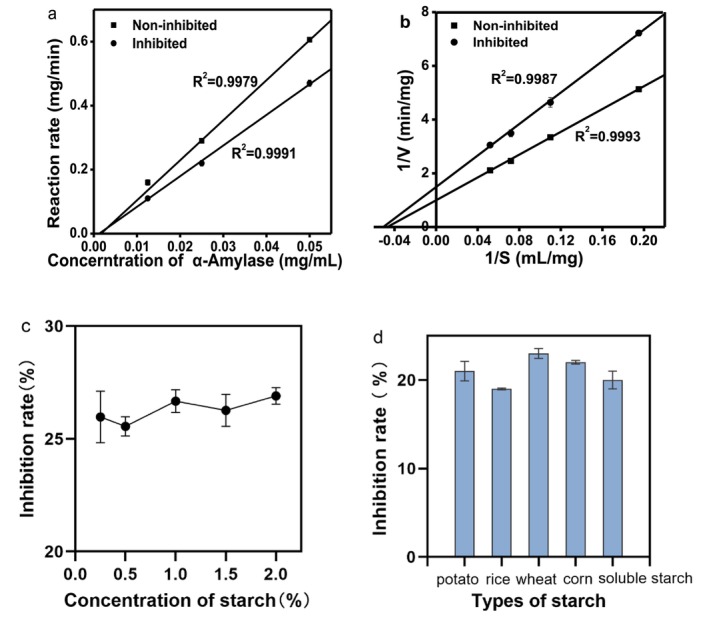
Inhibition type and starch inhibition type. (a) Inhibition kinetic diagram of α‐AI extract; (b) Lineweaver–Burk diagram of α‐AI extract; (c) effects of starch concentration on the inhibition of α‐AI; and (d) effects of starch breed (Starch concentration was 1%) on the inhibition of α‐AI. Error bars represent SD.

The type of inhibition demonstrated by enzyme inhibitors was intricately associated with their specific applications. Figure 2a presented the inhibition kinetics of α‐amylase by the crude extract of white kidney bean α‐AI. The rate of enzymatic hydrolysis for both uninhibited and inhibited α‐amylase approached the origin in a manner that suggested the inhibitory effect of the white kidney bean α‐AI on α‐amylase was reversible. Examination of the Lineweaver–Burk plot (Figure 2b) indicates that the intercepts on the horizontal axis for both uninhibited and inhibited α‐amylase are nearly identical, whereas the intercept on the vertical axis for inhibited α‐amylase is greater than that for uninhibited α‐amylase. This observation implies that the Michaelis constant (Km) for the α‐amylase hydrolysis reaction remains unchanged upon inhibition by the white kidney bean α‐AI crude extract, while the maximum reaction rate (Vmax) is reduced. The unchanged Km suggested that the inhibitor does not compete with the substrate for the active site, but instead binds to another region of the enzyme, altering its catalytic efficiency without affecting substrate affinity (Whiteley 2000). Therefore, it can be concluded that the inhibitory effect of the white kidney bean α‐AI crude extract on α‐amylase is indicative of non‐competitive inhibition.

Figure 2c illustrated the inhibitory effect curve of white kidney bean α‐AI on α‐amylase across varying starch concentrations. The augmentation of starch concentration (ranging from 0.25% to 2%) did not significantly alter the inhibition rate of white kidney bean α‐AI on α‐amylase (*p* > 0.05). The experimental data further confirmed that α‐AI from white kidney bean exhibited classical non‐competitive inhibition toward α‐amylase, where inhibitory efficacy remains unaffected by substrate concentration. The consistent performance under high substrate conditions highlights its suitability for real‐world applications where starch concentration may vary widely, such as in postprandial glucose modulation (Obiro et al. 2008). This substrate‐independent behavior underscores its potential utility in starch‐rich applications, including functional food production and glycemic management, as it maintains consistent enzyme suppression regardless of starch load, thereby laying a solid foundation for its translational development as a robust natural amylase inhibitor.

### 3.3 The Effect of Starch Type on the Inhibition of α‐AI From White Kidney Beans

Potato starch, rice starch, wheat starch, and corn starch are starch types widely present in people's daily diets. Figure 2d showed the inhibitory effect of white kidney bean α‐AI on α‐amylase under different starch types. Although the inhibitory effect of white kidney bean α‐AI on α‐amylase varies significantly (*p* < 0.05) when the starch type is different, these changes are not extremely significant (*p* > 0.01). The observed variation in inhibition may be attributed to differences in the structural properties of starch types, such as amylose/amylopectin ratio, granule morphology, and crystallinity, which can influence enzyme accessibility and binding efficiency (Magallanes‐Cruz et al. 2017). Despite these minor variations, the consistent inhibitory efficacy across common dietary starch sources supports the broad applicability of white kidney bean α‐AI as a universal amylase inhibitor, enhancing its potential for use in diverse food matrices and glycemic control strategies.

### 3.4 Effects of α‐AI on Body Weight and Lipid Metabolism

**FIGURE 3:**
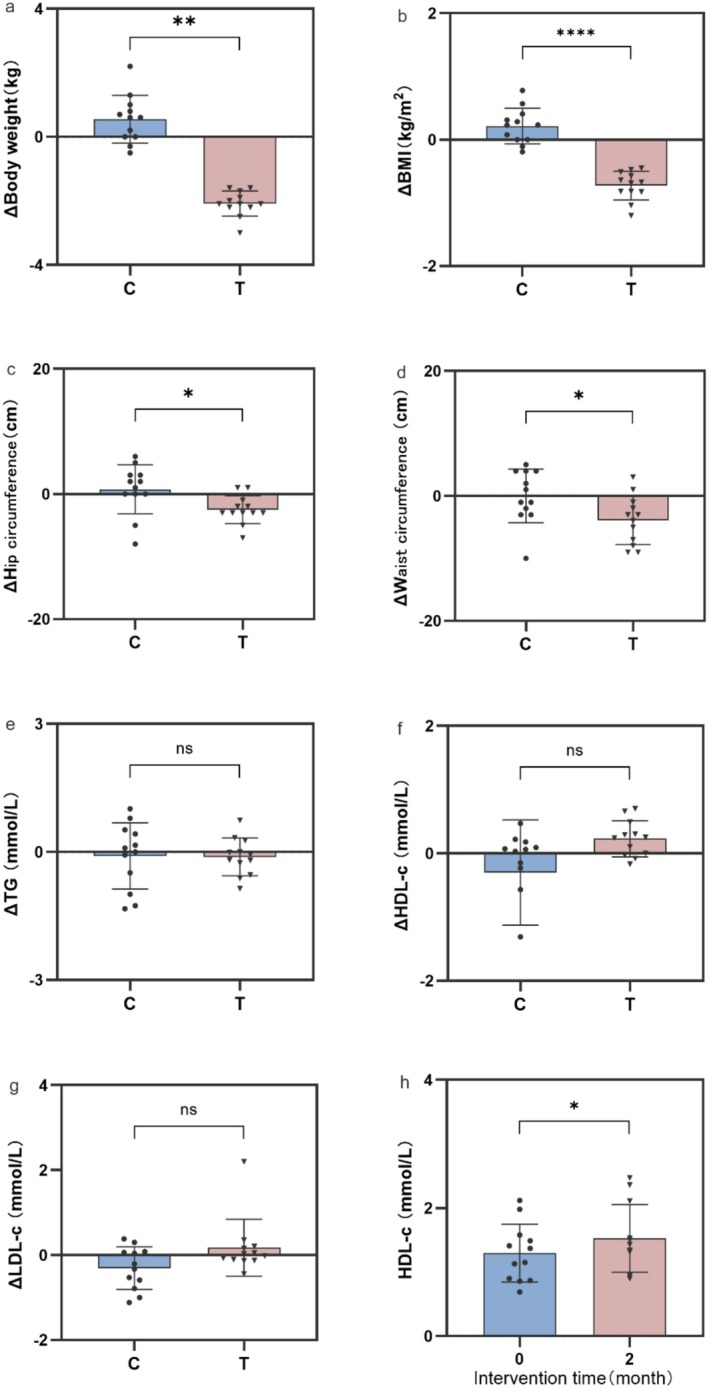
Effects of α‐AI on body weight and lipid metabolism. (a) Change of body weight; (b) change of BMI; (c) change of hip circumference; (d) change of waist circumference; (e) change of △TG; (f) change of △HDL‐c; (g) change of △LDL‐c; and (h) HDL‐c of test group before and after intervention. Error bars represent SD. The statistical symbols “*” defined as *p <* 0.05, “**” defined as *p <* 0.01, “****” defined as *p <* 0.000.

As shown in Figure 3a after taking the α‐AI for 8 weeks, the experimental group significantly reduced their weight by 2.08 ± 0.11 kg, while the control group increased by 0.55 ± 0.22 kg compared to before enrollment, indicating that the inhibitor can reduce carbohydrate absorption and achieve weight control. The results strongly supported the efficacy of α‐AI derived from white kidney bean as an intervention for weight management. This effect is primarily attributed to the inhibitor's ability to block the enzymatic action of α‐amylase, thereby reducing the hydrolysis of complex carbohydrates into absorbable monosaccharides. The subsequent decrease in carbohydrate absorption likely creates a negative energy balance, facilitating weight loss. This mechanism aligned with previous studies demonstrating that α‐AI supplementation can reduce postprandial glucose elevation and overall caloric intake, contributing to body weight control (Celleno et al. 2007).

Following an 8‐week intervention, the experimental group exhibited a BMI reduction of 0.73 ± 0.07 (Figure 3b), in marked contrast to the control group's increase of 0.216 ± 0.08, demonstrating a highly significant intergroup difference (*p <* 0.0001). Notably, the experimental group showed significantly greater reductions in both waist circumference (−4.2 ± 0.5 cm) and hip circumference (−3.1 ± 0.3 cm) compared to control measurements (Figure 3c,d). These findings indicate that α‐AI not only reduces overall weight but may also improve central obesity. Central obesity is closely linked to visceral fat accumulation, and its reduction is often associated with improved insulin sensitivity and decreased inflammation. Although this study did not directly measure fat distribution or metabolic parameters, the decline in waist‐hip ratio may indirectly reflect reduced visceral fat, which could be related to improvements in lipid metabolism.

Despite significant weight loss, no notable differences were observed in TG, HDL‐c, and LDL‐c levels between the control and experimental groups (Figure 3e–g). This limitation is compounded by the mechanism of action of α‐AI, which primarily reduces glucose absorption but may leave the intake of other nutrients—such as fats and proteins—unaffected, thereby weakening its overall impact on blood lipids. Furthermore, individual variability and the lack of strict dietary standardization pose additional challenges, as other sources of lipids in the diet could mask the effects of α‐AI, making it difficult to isolate its specific contribution to lipid profile changes.

Notably, HDL‐c levels were significantly increased (*p* < 0.05) within the experimental group when comparing pre‐ and post‐intervention measurements (Figure 3h). High‐density lipoprotein cholesterol (HDL‐C) level is a common clinical metric, as HDL is responsible for reverse cholesterol transport—carrying cholesterol from peripheral tissues to the liver for metabolism. Through this beneficial mechanism, HDL‐C helps reduce overall lipid levels, and its elevation is generally associated with a lower risk of cardiovascular disease (Kudinov et al. 2020). The observed elevation in HDL‐c may be mediated through multiple pathways. Recent evidence suggests that dietary and pharmacological interventions can enhance HDL functionality and concentration by promoting the expression of key transporters such as ABCA1 and ABCG1, which facilitate cellular cholesterol efflux (Barylski et al. 2014). Another potential mechanism involves the modulation of gut microbiota and increased production of SCFAs such as propionate and butyrate, which have been shown to upregulate hepatic HDL synthesis and promote reverse cholesterol transport (Han et al. 2021). Therefore, the increase in HDL‐c levels observed in this study further supports the potential cardiometabolic benefits of α‐AI supplementation beyond its established effects on weight management.

### 3.5 Variation of Structure of Gut Microbiota

**FIGURE 4:**
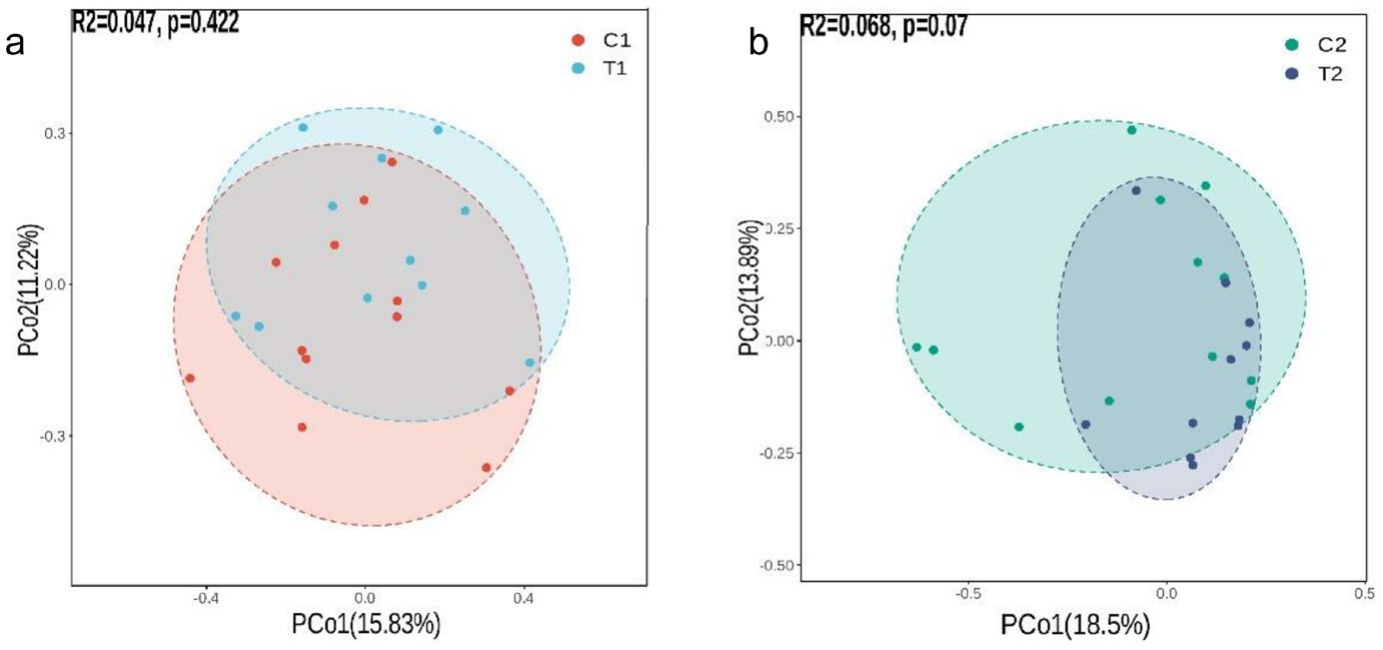
Changes of gut microbiota structure before and after intervention.

A significant alteration in the β diversity of the gut microbiota was observed in obese individuals following an 8‐week intervention with α‐AI, as indicated by the *p* value dropping from 0.398 to 0.028 (Figure 4). The significant shift in β diversity indicates that the α‐AI intervention considerably remodels the gut microbial community structure in obese individuals. β diversity serves as a crucial metric for assessing variations in microbial communities between samples, and the significant reduction in *p*‐values indicates that the post‐intervention changes in microbial community structure are statistically significant. The α‐AI may alter the nutritional environment in the gut by affecting carbohydrate digestion and absorption, thereby impacting the composition and function of the microbiota (Liu, Meng, et al. 2025). These structural shifts may underlie metabolic improvements associated with obesity, given the gut microbiota's pivotal role in energy harvest and fat storage.

### 3.6 The Screen of Key Bacteria in the Gut Microbiota Structure After Intervention

**FIGURE 5:**
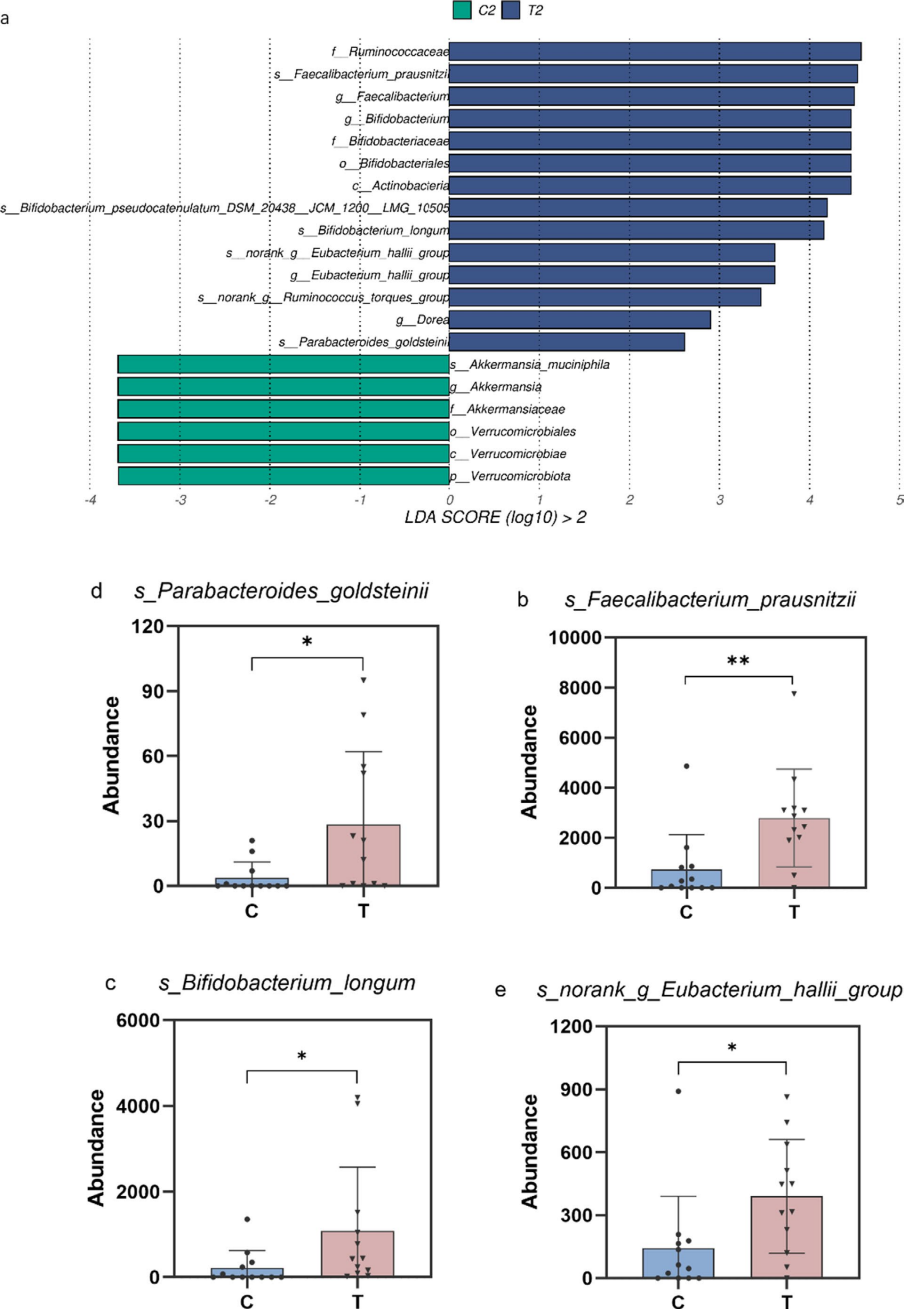
Differences in key bacteria after intervention. (a) Lefse analysis of control and test group after intervention; (b) abundance of *s_Faecalibacterium_prausnitzii*; (c) abundance of *s_Bifidobacterium_longum*; (d) abundance of *s_Parabacteroides_goldsteinii*; and (e) abundance of *s_norank_g_Eubacterium_hallii_group*. The statistical symbols “*” defined as *p <* 0.05, “**” defined as *p <* 0.01.

The enrichment of specific beneficial bacteria, such as *Bifidobacterium longum* , *Bacteroides ovatus* , and *Parabacteroides goldsteinii* , suggested a targeted prebiotic‐like effect of α‐AI (Figure 5). *B. longum* , a well‐documented probiotic, contributes to gut integrity, reduces inflammation, and modulates host energy metabolism via SCFA production (Salazar et al. 2009; Spaiser et al. 2015). SCFAs serve as both a crucial energy substrate for colonocytes and key regulators of systemic lipid metabolism and insulin sensitivity (Childs et al. 2014; Christensen et al. 2020). Similarly, the increased abundance of *Bacteroides ovatus* and *Parabacteroides goldsteinii* may enhance metabolic outcomes by facilitating complex carbohydrate breakdown and engaging in syntrophic interactions that support microbial diversity and ecosystem stability (Mary and Kapoor 2022; Vega‐Sagardía et al. 2023). Certain Bacteroides species are increasingly associated with improved weight control and lipid homeostasis (Christensen et al. 2020; Chung et al. 2020). It is important to note, however, that current evidence regarding the metabolic functions of these bacteria largely derives from animal or in vitro models; thus, their causal roles and mechanisms in humans require further clinical validation.

In conclusion, α‐AI appears to positively reshape the gut microbiota in obesity by selectively promoting beneficial genera like *Bifidobacterium* and *Bacteroides*, thereby contributing to improved metabolic health. This structural remodeling may be mediated through enhanced SCFAs production and strengthened gut barrier function. These findings position α‐AI as a potential modulator of host metabolism via the gut microbiome, offering novel therapeutic avenues for obesity and metabolic disorders. Nevertheless, limitations such as small sample size, short intervention duration, and lack of precise target identification should be acknowledged. Specifically, the use of 16S rRNA sequencing limited our analysis to genus‐level taxonomic changes and precluded insights into functional microbial pathways. Furthermore, the inferred role of SCFAs remains hypothetical in the absence of direct quantitative measurements. Future research should therefore integrate shotgun metagenomics to elucidate functional gene profiles and gas chromatography–mass spectrometry (GC–MS) to quantitatively assess SCFA concentrations. These approaches, combined with mechanistic assays, are essential to establish causal relationships within the α‐AI–microbiota–host metabolism axis.

### 3.7 The Dynamic Variation of Metabolic Pathway After the Intervention

**FIGURE 6:**
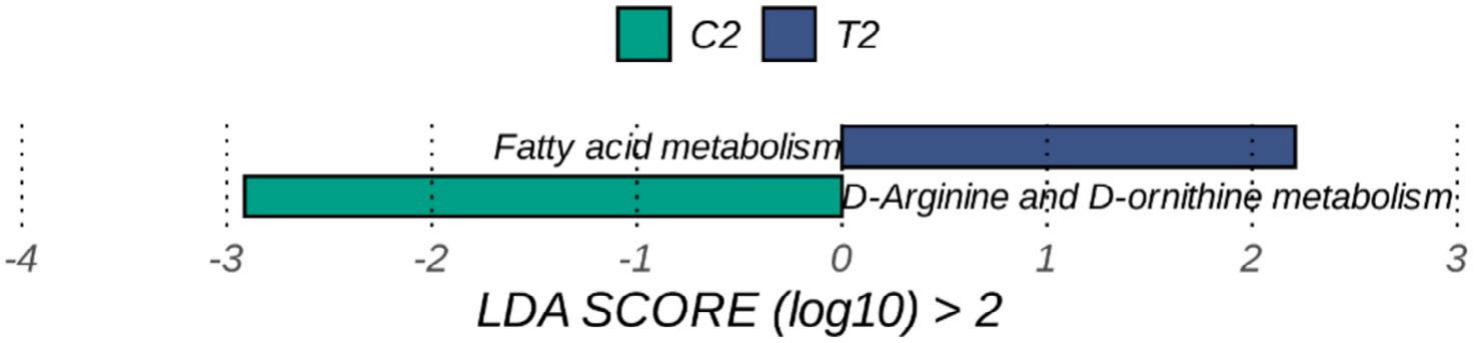
Differences in key metabolic pathways.

Illustrated in Figure 6, the LEfSe differential analysis identified significant alterations in fatty acid metabolism within the experimental group. These alterations primarily occurred in the metabolic pathways associated with SCFAs and long‐chain polyunsaturated fatty acids (LC‐PUFA). The study indicated that the increase in SCFAs was closely related to changes in the gut microbiota, particularly an increase in the abundance of probiotics such as *Bifidobacteria* and *Lactobacilli* (Nolan et al. 2020). These changes in microbial communities may enhance the production of SCFAs, thereby influencing the host's energy metabolism and fat storage. The relationship between fatty acid metabolism and obesity is complex and multifaceted. SCFAs, including acetic acid, propionic acid, and butyric acid, are produced in the gut through microbial fermentation of dietary fiber. They not only serve as an important energy source for intestinal epithelial cells but also play a significant role in regulating lipid metabolism and insulin sensitivity (Machate et al. 2020; Lin et al. 2012).

In addition, long‐chain polyunsaturated fatty acids (LC‐PUFAs) play a crucial role in regulating inflammation and lipid metabolism. Studies have shown that LC‐PUFAs can improve lipid metabolism by influencing gene expression in the liver and adipose tissue, reducing hepatic fat content and systemic inflammatory levels (Graf et al. 2019). In obese individuals, the metabolism of LC‐PUFAs may be influenced by gut microbiota; changes in specific bacterial populations, such as Bacteroides and Firmicutes, are closely related to the metabolism of LC‐PUFAs (Kundi et al. 2021).

Therefore, the α‐AI affects the composition and function of intestinal flora, thereby altering fatty acid metabolic pathways, which may be an important mechanism for its weight loss effect. This finding provides a new perspective for further research on the role of intestinal flora and fatty acid metabolism in obesity (Zhang et al. 2023). However, the exact signaling mechanisms and causal chain from microbial changes to fatty acid flux remain to be fully elucidated. Future studies using isotope tracing and metagenomic‐functional assays could help clarify these linkages.

## 4 Conclusion

This study systematically identified white kidney bean variety A10 as the most potent source of α‐amylase inhibitor (α‐AI), owing to its exceptional inhibitory activity and stability. The extracted α‐AI exhibited a non‐competitive inhibition mechanism, ensuring effective activity across a range of starch substrates, which underscores its potential for broad nutritional and therapeutic application. In a clinical setting, an 8‐week intervention using α‐AI derived from A10 led to significant reductions in body weight, BMI, waist circumference, and hip circumference in obese individuals. More importantly, this study revealed previously underexplored dimensions of α‐AI's action: it markedly modulated the gut microbiota composition, increasing the abundance of beneficial bacteria such as *Bifidobacterium* and *Bacteroides ovatus* . These microbial changes were correlated with shifts in host fatty acid metabolism, particularly in pathways related to short‐chain and long‐chain fatty acid synthesis and utilization.

The dual mechanisms of α‐AI, direct enzymatic inhibition of carbohydrate digestion and indirect regulation of the gut microbiome, highlight its multifactorial role in weight management. These findings provide a deeper scientific understanding of how α‐AI functions beyond mere enzyme inhibition, positioning it as a promising agent that integrates metabolic and macrobiotic modulation.

However, this study has several limitations that should be acknowledged. The clinical trial was conducted with a relatively small sample size and over a short‐term intervention period, which may affect the generalizability and long‐term applicability of the results. Although changes in microbial composition and metabolic pathways were observed, the exact causal mechanisms linking α‐AI intake, specific bacterial enrichment, and host metabolic improvements remain unclear. Furthermore, the study did not explore potential variability in individual responses based on dietary habits, genetic background, or baseline gut microbiota composition. These limitations point toward valuable directions for future research. Larger, longer term, and more diverse clinical cohorts are necessary to validate the sustainability and safety of α‐AI intervention. Mechanistic studies using germ‐free animal models, metagenomics, and metabolomics approaches could help elucidate the causal pathways underlying the observed microbiota–metabolism interactions. Additionally, personalized approaches considering individual microbiome profiles may enhance the efficacy of α‐AI‐based nutritional strategies.

The implications of this study are considerable. It not only supported the use of α‐AI as an effective natural intervention for obesity but also opened avenues for designing personalized nutritional strategies that target both host metabolism and gut ecosystem health. Future research should focus on elucidating the precise causal relationships between specific microbial changes and metabolic outcomes, as well as exploring the long‐term efficacy and safety of α‐AI supplementation in larger and more diverse populations.

## Author Contributions

**Jiai Yan:** data curation (lead), formal analysis (lead), writing – original draft (equal), writing – review and editing (equal). **Jianguang Zhao:** investigation (equal), methodology (equal). **Pamila Naizemuding:** project administration (equal), supervision (equal). **Wei Zhao:** writing – review and editing (equal). **Jing Sun:** conceptualization (equal), data curation (equal), software (equal), validation (equal). **Yingyu Wang:** formal analysis (equal), funding acquisition (equal). **Ju Yang:** data curation (equal), formal analysis (equal), software (equal), validation (equal). **Dan Li:** formal analysis (equal), funding acquisition (equal), validation (equal). **Feng Zhang:** funding acquisition (equal). **Hong Cao:** project administration (equal).

## Conflicts of Interest

The authors declare no conflicts of interest.

## Data Availability

The data that support the findings of this study are available on request from the corresponding author. The data are not publicly available due to privacy or ethical restrictions.
